# Understanding consumers’ continuance intention to watch streams: A value-based continuance intention model

**DOI:** 10.3389/fpsyg.2023.1073301

**Published:** 2023-03-01

**Authors:** Xiaoyun Jia, Yan Pang, Bingqi Huang, Feng Hou

**Affiliations:** ^1^School of Politics and Public Administration, Institute of Governance, Shandong University, Qingdao, China; ^2^School of Mathematical and Computational Sciences, Massey University, Auckland, New Zealand; ^3^School of Logistics and Transportation, Central South University of Forestry and Technology, Changsha, China; ^4^Guanghua School of Management, Peking University, Beijing, China

**Keywords:** continuance intention of watching, behavioral intention, live streaming, ECT, V-ECM, post-adoption behavior

## Abstract

**Introduction:**

Live stream-watching has become increasingly popular worldwide. Consumers are found to watch streams in a continuous manner. Despite its popularity, there has been limited research investigating why consumers continue to watch streams. Previously, the expectation-confirmation theory (ECT) has been widely adopted to explain users’ continuance intention. However, most current ECT-based models are theoretically incomplete, since they only consider the importance of perceived benefits without considering users’ costs and sacrifices. In this paper, we propose a *value-based continuance* intention model (called V-ECM), and use it to investigate factors influencing consumers’ continuance intention to watch streams.

**Methods:**

Our hypotheses were tested using an online survey of 1,220 consumers with continuance stream-watching experiences.

**Results:**

Results indicate that perceived value, a process of an overall assessment between users’ perceived benefits and perceived sacrifices, is proved to be a better variable than perceived benefits in determining consumers’ continuance watching intention. Also, compared with other ECT-based models, V-ECM is a more comprehensive model to explain and predict consumers’ continuance intention.

**Discussion:**

V-ECM theoretically extends ECT-based studies, and it has potential to explain and predict other continuance intentions in online or technology-related contexts. In addition, this paper also discusses practical implications for live streaming platforms with regards to their design, functions and marketing.

## 1. Introduction

The live streaming industry has been growing explosively and becoming popular worldwide (Jia et al., 2021). The time that consumers spend on stream-watching has been increasing. The live streaming watch time has increased by 250% (Dacast, 2022). The total number of hours watched have been increasing steadily from 4.83 billion in the first quarter of 2020 to 8.26 billion in the fourth quarter of 2020 (Statista, 2022). As of late 2020, consumers’ weekly stream-watching time has increased 59% compared with the watching hours in 2018, reaching 16 h (Restream, 2020).

With the rise in time spent on stream-watching, it becomes important to understand why consumers watch live streams and continue to watch. The factors influencing consumers’ initial intention of watching streams has already been well investigated (Hamilton et al., 2014; Gros et al., 2017; Sjöblom and Hamari, 2017; Long and Tefertiller, 2020). However, consumers’ continuance stream-watching intention is still not fully understood. It has been widely accepted that, theoretically, initial intention and continuance intention are different. Initial intention is a pre-adoption intention influenced by consumers’ indirect experiences, while continuance intention is a post-acceptance intention mainly influenced by consumers’ direct experiences (Bhattacherjee, 2001b). Likewise, the initial intention of watching differs from continuance intention of watching. Initial intention of watching can explain why consumers watch live streams for the first time, but it fails to illustrate why they continue to watch. Hence, there is a need to theoretically study and explain consumers’ continuance intention of watching streams. In practice, understanding consumers’ continuance intention of watching is also important. Firstly, retaining existing consumers only cost one-fifth compared with acquiring new consumers (Hossain and Quaddus, 2012). Hence, understanding the factors influencing consumers’ continuance use could help retain existing consumers and save money. Also, the eventual success of an industry is, to a great extent, determined by consumers’ continuance use (Bhattacherjee, 2001b). So is the live streaming industry.

In literature, expectation-confirmation theory (ECT) has been widely used to explain customers’ continuance intention. In particular, Bhattacherjee’s *post-acceptance model of information system (IS) continuance* (Bhattacherjee, 2001b) is the most widely accepted expectation-confirmation model (ECM). Most ECT-based continuance intention studies have been built on this model. However, most ECMs are incomplete in theory since they only take into account that consumers’ perception of expected benefits may change over time (Bhattacherjee, 2001b), but fail to consider that their perception of losses and costs may also change over time in their continuance uses. In other words, most current models only suit in an ideal situation in which consumers perceive benefits only without any losses or costs in their continuance use of a product or service. This leads to an inappropriate proposition that no matter how high the losses and costs of use that consumers may perceive, they would re-use the product or service that they were satisfied with, as long as they still perceive some benefits of use. We believe that consumers tend to practically compare their perceived benefits with their perceived costs and losses of use, to calculate if their continuance use is worthwhile. If consumers’ perceived benefits are higher than their perceived losses and costs, they are more likely to continue to use the product or service. However, if consumers perceive more losses and costs than benefits, continuance use is unlikely to happen. In addition, this benefit-cost analysis is not one-off, but continuous. This means consumers’ overall perception may be different over time.

So far, only a few studies (Lin et al., 2012; Yen et al., 2013; Hsu and Lin, 2015) have tried to include this benefit-cost analysis in their ECMs. However, benefits, costs or values in these models have not been properly defined. They have never been viewed as the constructs in their ECMs, but only as categories of variables.

We propose a *value-based continuance intention model*, named V-ECM. In this model, we include a new comprehensive construct, perceived value, to theoretically improve ECT. This new construct represents a dynamic benefit-cost assessment, indicating that consumers will continuously assess and compare their perceived benefits and perceived sacrifices. We also involve two pivotal factors, perceived ease of use and subjective norms, to enhance the explanation ability of continuance intention.

Compared with other countries, the live streaming industry in China is the biggest with regard to the revenue generated and the number of consumers (Restream, 2020; Xinhuanet, 2020). Therefore, we test our proposed V-ECM using data collected from the Chinese stream-watching consumers.

The remainder of the paper is organized as follows. Section 2 provides the background literature; Sections 3 and 4 describe our hypotheses and methods; Section 5 details the data analysis results; Section 6 presents a discussion of the results and their implications; and finally, Section 7 analyses the limitations of this study and provides suggestions for future research.

## 2. Theoretical background

### 2.1. The post-acceptance model of IS continuance

The most prominent theory employed to explain consumers’ post-adoption behavior is the expectation-confirmation theory (ECT), which indicates that consumers’ continuance intention is mainly determined by their satisfactions with prior product/service uses (Oliver, 1980; Bhattacherjee, 2001a). It originated from consumer behavior research but has later been applied in multiple disciplinary domains (Bhattacherjee, 2001b).

The original ECT model represents the continuance adoption processes as follows: initially, before adopting a product or service, consumers produce an expectation of a product or service. Then, consumers adopt the product or service, and they perceive its performance after use. Next, consumers compare their original expectation and perceived performance, and estimate whether their expectation is confirmed. A high level of confirmation leads to satisfaction, which has a positive influence on consumers’ continuance intention (Oliver, 1980; Bhattacherjee, 2001b).

**FIGURE 1:**
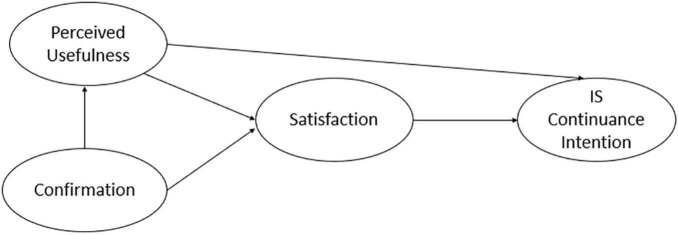
The post-acceptance model of IS continuance.

Later, Bhattacherjee (2001b) noticed: (i) the original model could not explain why some initial consumers discontinued their use; (ii) the original model did not consider the emergence of consumers’ psychological motivations after consumers’ initial use of a product or a service. He then modified the model and extended it to a *post-acceptance model of IS continuance* (as shown in Figure 1). The processes of the modified ECT are as follows: before consumers use a product or service, they tend to form an expectation. Then the original expectation is compared with consumers’ perceived performance after consumers adopt the product or service, and this comparison determines how much the consumers’ expectation is confirmed. This confirmation also influences consumers’ post-adoption expectation (presented as perceived usefulness in the *post-acceptance model of IS continuance*) which may change with time. On the one hand, consumers’ perception of usefulness could directly affect their re-adoption intention. On the other hand, consumers’ confirmation level and perceived usefulness, in turn, influence their satisfaction which mediates their re-adoption intention (Bhattacherjee, 2001b).

The *post-acceptance model of IS continuance* has been widely used to investigate consumers’ continuance intention in different contexts, such as online banking, electronic commerce, mobile data services, the web portal context, blogs, Internet protocol television, paid mobile apps, and mobile instant messaging (Bhattacherjee, 2001a,b; Lin et al., 2005, 2012; Kim, 2010; Shiau et al., 2011; Hsu and Lin, 2015; Oghuma et al., 2016).

Similarly, continuance stream-watching is a post-adoption behavior. Consumers decide to continue watching streams, at least in part, because they are satisfied with their prior watching experiences. Therefore, it is feasible to apply ECT to explain consumers’ continuance intention of watching streams.

### 2.2. Perceived usefulness and perceived value

In the post-acceptance model of IS continuance, perceived usefulness only focuses on consumers’ cognitive assessment of the adoption (Bhattacherjee, 2001b,Mamun et al., 2020). Many scholars have argued that perceived usefulness is not adequate, and they suggest to include non-utilitarian benefits (Delone and Mclean, 2004; Oghuma et al., 2016) in the model. Mamun et al. (2020) hence use perceived benefits to replace perceived usefulness to include additional advantages over usefulness.

Even considering additional advantages and benefits, as presented before, ECT is still incomplete in theory since it omits consumers’ perception of losses and costs. Hence, current ECT only works in an ideal situation where consumers only perceive benefits without any losses or costs in their continuance use of a product or service, leading to an inappropriate proposition that no matter how high the losses and costs of use that consumers’ may perceive, they would continue to adopt as long as they still perceive some benefits from adoption. Hence, there is a need to revise current ECT.

Perceived value is a concept which can be adopted to ECT. It is originally used in an initial adoption study (Kim et al., 2007). It refers to the overall trade-off of perceived benefits (PB) and perceived sacrifices (PS) (Kim et al., 2007). It can be formulated as:


PV=PB-PS

Where perceived benefits were consisted of usefulness and enjoyment, while perceived sacrifices were made up of technicality and perceived fee (Kim et al., 2007). Notably, perceived benefits and perceived sacrifices were categories of variables, rather than the constructs in the model.

Also, the components of perceived benefits and perceived sacrifices are measured differently in different contexts. Perceived benefits were measured by personalization, high quality, content richness, and value-added services, and perceived sacrifices were made up of comprised of perceived fee, change of viewing habits, technicality, and knowledge of alternatives in the Internet protocol television (IPTV) context (Lin et al., 2012). We intend to use perceived value which represents the benefit-cost analysis to replace perceived usefulness, since perceived value is a more theoretically complete and comprehensive variable.

## 3. Research model and hypotheses

### 3.1. The baseline model

In our model, perceived benefits and perceived sacrifices are defined to fit broader contexts. Perceived benefits in our model are defined as any advantages or gains from using or continuance using a product or service, no matter whether utilitarian, hedonic, or social, such as entertainment, monetary benefits, emotional benefits, psychological benefits and social benefits. Perceived sacrifices in our model are defined as any losses or costs from using or continuance using a product or service, such as monetary loss, time loss, loss of fame, or efforts spent in using. Overall, both perceived benefits and perceived sacrifices can be monetary or non-monetary, extrinsic or intrinsic, tangible or intangible. Perceived value in our model thus refers to consumers’ overall perception of using or continuance using a product or service after considering both its benefits and their sacrifices. Therefore, perceived value can be monetary or non-monetary, extrinsic or intrinsic, tangible or intangible as mentioned above. In this study, perceived value refers to consumers’ overall perception of live stream-watching or continuance watching after assessing the benefits and sacrifices they perceive. As Bhattacherjee’s *post-acceptance model of IS continuance* (Bhattacherjee, 2001b) is our baseline model, after replacing perceived usefulness with our perceived value, we set forth the following hypotheses:

**H1**: Confirmation has a positive effect on perceived value.

**H2**: Perceived value has a positive effect on satisfaction.

**H3**: Satisfaction has a positive effect on continuance intention of watching.

**H4**: Confirmation has a positive effect on satisfaction.

**H5**: Perceived value has a positive effect on continuance intention of watching.

### 3.2. Additional factors

Perceived ease of use has been demonstrated to be a useful predictor of technology adoption intention in the studies of consumers’ acceptance of computer technology (Davis, 1989), e-portfolios (Abdullah et al., 2016), web-based learning platforms (Sánchez and Hueros, 2010), smartphone usage (Joo and Sang, 2013), continuance usage of a fitness app (Beldad and Hegner, 2018), and continuance IT usage (Thong et al., 2006). It refers to the perception that the use of a system/application is easy and convenient (Davis, 1985).

Continuance stream-watching is also a technology-related activity. Live streaming is a new medium, and its use is different from traditional media such as television and newspapers. Streams can be accessed through live streaming websites or live streaming apps. Therefore, similar to other technology adoption intentions (Davis, 1989; Joo and Sang, 2013), continuance stream-watching intention may be influenced by perceived ease of use as well. Hence, we propose:

**H6**: Perceived ease of use has a positive effect on continuance intention of watching.

Perceived ease of use has also been validated to positively impact online consumers’ satisfaction in the prior literature (Dalcher and Shine, 2003; Joo et al., 2011, 2018; Tu et al., 2012; Amin et al., 2014; Shah and Attiq, 2016; Lin et al., 2017). It indicates that consumers are more likely to develop satisfaction when they perceive that the online system/application is easy to use. Hence, we hypothesize:

**H7**: Perceived ease of use has a positive effect on satisfaction.

In addition, perceived ease of use has been found to correlate to perceived usefulness and/or perceived playfulness in continuance intention studies (Thong et al., 2006; Wangpipatwong et al., 2008; Kim et al., 2009; Zhou, 2011; Yoon et al., 2015; Beldad and Hegner, 2018; Joo et al., 2018). As perceived value is a broader variable used to replace perceived usefulness and perceived playfulness, we predict that:

**H8**: Perceived ease of use has a positive effect on perceived value.

Subjective norms (also known as “social norms”) have been identified as a critical factor in the previous continuance intention studies. Subjective norms refer to “the perceived expectations of specific referent individuals or groups, and the person’s motivation to comply with those expectations” (Fishbein and Ajzen, 1975).

Prior quantitative studies demonstrated that subjective norms have a direct positive influence on continuance intention (Lee, 2010; Kim, 2011; Chen et al., 2012; Chang et al., 2014; Zhou and Li, 2014; Bhattacherjee and Lin, 2015; Mouakket, 2015; Yoon and Rolland, 2015; Liébana-Cabanillas et al., 2021). Also, in a recent qualitative study of live streaming, subjective norms were found to affect consumers’ stream-watching behavior. Consumers were found to prefer watching streams recommended by their important others (Jia et al., 2020). We propose that subjective norms also affect consumers’ continuance intention of watching. This leads to the following hypothesis:

**H9**: Subjective norms have a positive effect on continuance intention of watching.

The relationship between subjective norms and satisfaction has not been investigated in much depth in the continuance intention studies. The existing literature shows (or partially demonstrates) that subjective norms positively influence satisfaction (Hsu and Chiu, 2004; Chen et al., 2012). Hence, we predict that:

**H10**: Subjective norms have a positive effect on consumers’ satisfaction.

Subjective norms have also been found to influence perceived usefulness in the technology-related studies (Teo, 2009, 2010; Weiz et al., 2016). Furthermore, subjective norms were shown to have a positive impact on perceived usefulness in a meta-analysis study (Schepers and Wetzels, 2007). Again, as mentioned before, in this study, perceived value is a broader variable and is used to replace perceived usefulness. Therefore, we propose:

**H11**: Subjective norms have a positive effect on perceived value.

**FIGURE 2:**
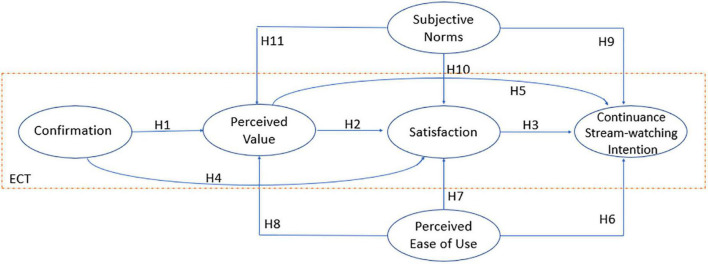
Proposed V-ECM.

Based on the reasoning and hypotheses formulated above, our proposed conceptual V-ECM is shown in Figure 2.

## 4. Research methods

### 4.1. Participants

As presented before, the live streaming industry in China is bigger compared with other countries considering the revenue generated and the number of consumers (Restream, 2020; Xinhuanet, 2020). Hence, I subjects of this study were Chinese consumers with continuance stream-watching experiences who were 18 years or older at the time of the survey. Data were collected through Wenjuanxing,^1^ one of the most popular Chinese online survey tools. There was a total of 1,220 final valid responses after deleting 264 invalid responses. The invalid responses include (i) 43 responses with all the same answer, (ii) 98 responses answered within an extremely short response time (60 s), and (iii) 123 responses with conflicting views. The age of the subjects ranged from 18 to 60 with a median age of 25. In particular, 66.2% of the subjects were under 30 years old, which is consistent with the survey indicating that a majority of live streaming consumers are not older than 30 years old (Statista, 2020). The details of the demographic information of the subjects are shown in Table 1.

**TABLE 1:** Demographic information of the subjects.

Items	Categories	Frequency	Percent
Age	18–24	559	45.8
	25–30	249	20.4
	31–35	163	13.4
	36–40	147	12
Gender	Over 41	102	8.4
	Male	522	42.8
	Female	455	37.3
Occupation	Other or not disclosed	243	19.9
	Students	500	41
	Professionals	576	47.2
	Unemployed or retired	144	11.8
Marital status	Single	496	40.7
	In a relationship	396	32.5
	Married or partnership	328	26.9

The most popular stream category among the participants was entertainment (such as singing, dancing), accounting for 68%. Other popular categories included gaming (47%), product-selling (31.3%), education and knowledge sharing (27.5%), and E-sports (24.6%).

### 4.2. Measurement development

In this study, six constructs were measured including confirmation, perceived value, satisfaction, subjective norms, perceived ease of use, and continuance intention of watching. Constructs were surveyed with items adapted from pre-validated measures. Each adapted item was modified to fit the live streaming context.

A 7-point Likert scale from 1 (strongly disagree) to 7 (strongly agree) was adopted to measure the participants’ attitudes. All of the items were reviewed by four academic experts and six senior doctoral candidates before implementation. Items that were questioned in the review process were modified. A pilot survey (*N* = 102) was subsequently conducted. All of the items were tested and showed satisfactory validity and reliability. The operationalization of the constructs is detailed in Table 2, and the items of the constructs are presented in the Appendix.

**TABLE 2:** Operationalization of the constructs.

Constructs	Operational definition	References
Confirmation	Consumers’ perception of the congruence between expected stream-watching and actual performance	Bhattacherjee, 2001b
Perceived Value	Consumers’ overall perception of stream-watching or continuance stream-watching after considering both benefits/advantages and sacrifices/costs. The overall perception can be monetary or non-monetary, extrinsic or intrinsic, tangible or intangible, etc	Kim et al., 2007
Satisfaction	Consumers’ feelings about prior experience of watching live streams	Bhattacherjee, 2001b
Perceived Ease of Use	Consumers’ perception of being free of effort when watching live streams	Venkatesh and Davis, 1996
Subjective Norms	Consumers’ perception of approval or disapproval of watching live streams from important others	Fishbein and Ajzen, 1980
Continuous Intention of Watching	Consumers’ intention to continue watching live streams	Bhattacherjee, 2001b

## 5. Data analysis and results

Our study used structural equation modelling (SEM) to test the measurement model and assess the hypotheses. SEM is a powerful statistical research technique for model-testing involving multiple-item constructs (Jöreskog and Sörbom, 1993). We adopted the method of maximum likelihood, the most widely used approach in SEM studies (Hair et al., 1998). SPSS 26.0 and Mplus were used for data analysis.

### 5.1. Measurement model

The goodness-of-fit of the model was measured using several fit metrics (Browne and Cudeck, 1993; Hoe, 2008; West et al., 2012; Di Leo and Sardanelli, 2020) as presented in Table 3. Our model met all other criteria, demonstrating a good model fit.

**TABLE 3:** Measurement model.

Index	χ^2^/df	*p*	TLI	CFI	RMSEA
Value	1.736	0.000	0.983	0.986	0.025
Level of acceptance	<3	<0.05	≥0.95	≥0.95	≤0.06

The scales were tested via confirmatory factor analysis (CFA), with the results presented in Tables 4, 5. Factor loadings and average variance extracted (AVE) were assessed to check the convergent validity. Each item loading on the correct factor was higher than 0.6, and every AVE exceeded 0.5 (shown in Table 4), demonstrating acceptable convergent validity (Bagozzi and Yi, 1988). Also, all the square roots of the AVEs exceeded their corresponding correlation coefficients (shown in Table 5), indicating satisfactory discriminant validity (Fornell and Larcker, 1981). Cronbach’s alpha (CA) and Composite Reliability (CR), measures of internal consistency, were tested to assess the model’s reliability. Table 4 shows that all the values of CA and CR exceeded 0.7, revealing good reliability (Fornell and Larcker, 1981).

**TABLE 4:** Scale properties.

Constructs	Items	Factor loadings	AVE	Composite reliability	Cronbach’s alpha
Confirmation	CO1	0.736	0.570	0.799	0.798
	CO2	0.791			
	CO3	0.737			
Perceived value	PV1	0.736	0.589	0.811	0.811
	PV2	0.761			
	PV3	0.804			
Satisfaction	SA1	0.772	0.654	0.883	0.883
	SA2	0.825			
	SA3	0.811			
	SA4	0.826			
Subjective norms	SN1	0.798	0.646	0.845	0.844
	SN2	0.851			
	SN3	0.760			
Perceived ease of use	PEoU1	0.810	0.580	0.803	0.799
	PEoU2	0.803			
	PEoU3	0.658			
Continuance intention of watching	CWI1	0.971	0.711	0.877	0.859
	CWI2	0.607			
	CWI3	0.907			

**TABLE 5:** Correlation matrix.

	CO	PV	SA	Sn	PEoU	CWI
CO	**0.755**					
PV	0.380	**0.767**				
SA	0.266	0.288	**0.809**			
SN	0.164	0.253	0.191	**0.804**		
PEoU	0.224	0.137	0.249	0.094	**0.762**	
CWI	0.202	0.331	0.365	0.268	0.272	**0.843**

### 5.2. Structural model

**FIGURE 3:**
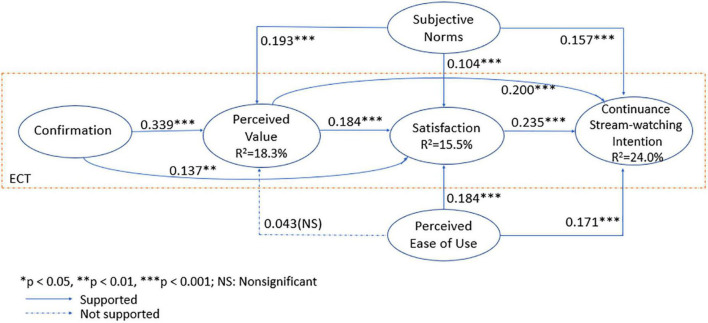
Structural equation model results for V-ECM.

All but one of the proposed hypotheses were supported. All the supported paths in the model were significant at *p* < 0.01. Figure 3 presents the results of the structural model. The results show that both confirmation (β = 0.339, *p* < 0.001) and subjective norms (β = 0.193, *p* < 0.001) had positive impacts on perceived value. Hence, H1 and H11 were supported. However, perceived ease of use (β = 0.043, *p* > 0.05) was not found to have a significant effect on perceived value. Hence, H8 was rejected. The results explained 18.3% of the variance in perceived value. Confirmation (β = 0.137, *p* = 0.001) was also found to have a positive effect on satisfaction. In addition, perceived value (β = 0.184, *p* < 0.001), subjective norms (β = 0.104, *p* < 0.001) and perceived ease of use (β = 0.184, *p* < 0.001) were all found to positively influence satisfaction. Hence, H4, H2, H10, and H7 were supported. The results explained 15.5% of the variance in satisfaction. Furthermore, satisfaction (β = 0.235, *p* < 0.001), perceived value (β = 0.200, *p* < 0.01), perceived ease of use (β = 0.171, *p* < 0.001), and subjective norms (β = 0.157, *p* < 0.001) were all found to have positive impacts on continuance intention of watching. Hence, H3, H5, H6, and H9 were supported. The results explained 24% of the variance in continuance intention of watching. In particular, satisfaction was the strongest indicator influencing continuance intention of watching. Table 6 details the main findings of our study.

**TABLE 6:** Summary of findings.

Hypothesis	Path	Coefficient*	Results
H1	Confirmation ⇒ Perceived value	0.339	Accepted
H2	Perceived value ⇒ Satisfaction	0.184	Accepted
H3	Satisfaction ⇒ Continuance intention of watching	0.235	Accepted
H4	Confirmation ⇒ Satisfaction	0.137	Accepted
H5	Perceived value ⇒ Continuance intention of watching	0.200	Accepted
H6	Perceived ease of use ⇒ Continuance intention of watching	0.171	Accepted
H7	Perceived ease of use ⇒ Satisfaction	0.184	Accepted
H8	Perceived ease of use ⇒ Perceived value	0.043 (NS)	Rejected
H9	Subjective norms ⇒ Continuance intention of watching	0.157	Accepted
H10	Subjective norms ⇒ Satisfaction	0.104	Accepted
H11	Subjective norms ⇒ Perceived value	0.193	Accepted

*All accepted hypotheses were supported by path coefficients significant at *p* < 0.01.

## 6. Discussion

### 6.1. Conclusion and discussion

In this paper, we propose V-ECM where we involve a practical process of trade-off between consumers’ perceived benefits and perceived sacrifices. Our proposed V-ECM fills in the theoretical gaps in current ECMs. Through re-defining perceived benefits, perceived sacrifices and perceived value, making perceived value as a pivotal generalized construct rather than a category in the model, and adding two additional effective variables (perceived ease of use and subjective norms), our proposed V-ECM works as a generalized model which can properly explain and predict continuance intention of watching, and has the potential to predict continuance intention in broad contexts.

This study theoretically improves ECT. Most previous studies only focus on consumers’ perceived benefits. This leads to an inappropriate proposition that consumers would continue their use of a product or service as long as they were satisfied with their previous use, even if their perceived costs and sacrifices of use became very high over time. In comparison, our proposed V-ECM can fix this deficiency by replacing perceived usefulness (or perceived enjoyment or other similar related variables) with perceived value which can reflect both perceived benefits and perceived sacrifices. In our model, for the first time, perceived value is measured as a vital construct, rather than a category of variables in continuance studies. This replacement of the construct makes V-ECM a better and more comprehensive model to explain and predict continuance intention. Overall, our V-ECM stresses that when consumers continue to use a product or service, they will continuously compare their perceived benefits and perceived sacrifices according to their practical circumstances. This trade-off (presented as perceived value) is one of the key determinants of their continuance intention.

In addition, in our model, perceived value, perceived benefits and perceived sacrifices have been provided with new comprehensive definitions. Moreover, additional factors (perceived ease of use and subjective norms) are included in V-ECM and proved to be important predictors in influencing consumers’ continuance intention, which extends ECT studies as well. Our proposed V-ECM explains well why consumers continue to watch live streams. As demonstrated by our V-ECM, besides satisfaction which has been validated by many previous ECT-based studies, perceived value, subjective norms, and perceived ease of use have direct influences on continuance intention of watching. Also, V-ECM is a more generalized model which may be applied to explain and predict continuance intentions in other online or technology-related contexts, such as continuance intention of watching short videos and continuance intention of using smartphone apps.

### 6.2. Implications

The findings of our investigation have both theoretical and practical implications.

In our study, consumers’ confirmation is found to have a positive association with perceived value and satisfaction. This suggests that for live streaming platforms, it would be important to manage consumers’ expectations and to exceed them. For example, platforms should let consumers know upfront what services that the platforms can provide, without exaggerating or misleading, in order to remove consumers’ uncertainty and hence confirm their expectations.

In our model, perceived value has been confirmed to be an important determinant of continuance watching intention, suggesting that consumers will continuously assess and compare the benefits that they can get and the sacrifices that they experience from watching live streams. If they perceive positive value (i.e., consumers perceive more benefits than sacrifices), they are more likely to perceive high satisfaction and continue to watch. However, if consumers’ perception of value is negative (i.e., they perceive fewer benefits than sacrifices), their satisfaction is likely to be lowered and their continuance intention of watching is likely to be hindered. Theoretically, it is the first time that perceived value has been found to work in the continuance intentions.

TIe implications are that live streaming platforms should provide streams with good quality and high value, which would promote consumers’ perceived usefulness, enjoyment and social benefits to trade off their perceived sacrifices from watching.

Satisfaction has been proved to be the most influential precedent in our study, which is consistent with most of the previous ECT-based studies (Bhattacherjee, 2001a,b; Lin et al., 2005, 2012, 2014; Shiau et al., 2011; Chang and Zhu, 2012; Zhang et al., 2015; Oghuma et al., 2016). Satisfaction has been found to work as a significant mediator as well. Satisfaction can not only mediate the relationship between perceived ease of use and continuance intention of watching as mentioned above, but also mediate the relationship between subjective norms and continuance intention, the relationship between confirmation and continuance intention of watching, and the relationship between perceived value and continuance intention. Hence, the importance of satisfaction is highlighted.

Hence, live streaming platforms should increase consumers’ satisfaction from many different aspects including providing personalized services, solving consumers’ complaints on time, providing opportunities to surprise and delight consumers.

Our study indicates the significant impact of subjective norms on continuance intention. Perceived value in our model is for the first time confirmed to be influenced by subjective norms in the continuance intention study. Our results also confirm the correlation relationship between subjective norms and satisfaction, which has not been investigated in much depth before. In addition, our results reveal that subjective norms not only have a positive influence on consumers’ intention of watching as confirmed before (Jia et al., 2020), but also positively affect consumers’ continuance intention of watching. In other words, subjective norms can affect consumers’ intention in any phases of watching.

According to these findings, live streaming platforms could make use of the effect of celebrity endorsement. It may also work if live streaming platforms could invite and encourage more celebrities, teachers, or politicians to broadcast on their platforms, since these people are more likely to be consumers’ important others for normative purposes.

Perceived ease of use was also found to have a considerable influence in our model. Our finding of the relationship between perceived ease of use and satisfaction is consistent with previous related studies (Dalcher and Shine, 2003; Joo et al., 2011, 2018; Tu et al., 2012; Amin et al., 2014; Shah and Attiq, 2016; Lin et al., 2017). In terms of the controversy over whether perceived ease of use is a factor impacting consumers’ continuance intention over decades, our study reveals that perceived ease of use can affect consumers’ continuance intention of watching in both direct and indirect (mediated by satisfaction) ways in the live streaming realm. Our findings are partially consistent with previous continuance intention studies (Gefen and Straub, 2000; Roca et al., 2006; Roca and Gagné, 2008; Wangpipatwong et al., 2008; Joo et al., 2018).

Based on our findings, live streaming platforms could therefore modify and improve their websites, apps, interfaces, and functions, to make them easier to access and use in order to increase consumers’ satisfaction and continuance intention of watching. However, our results also show that there is no correlation between perceived ease of use and perceived value. As our research is the first attempt to investigate their relationship, more studies are encouraged to test if they are correlated.

## 7. Limitations and future work

Direct generalization of our findings to countries with different cultural backgrounds may not be proper. In this study, the participants are the Chinese live streaming consumers. The results demonstrate that our proposed model works well in the Chinese culture. However, different cultural backgrounds may result in different results. Therefore, future studies may investigate the antecedents of consumers’ continuance intention of watching streams in different countries or cultural backgrounds, and compare their results with our study. For example, comparing Chinese and American consumers (e.g., Twitch consumers).

Theoretically, our model has the potential to be applied in other technology and online-related continuance intentions as well, for example, continuance watching of short videos. Hence, future related work could test V-ECM in different contexts.

## Data availability statement

The raw data supporting the conclusions of this article will be made available by the authors, without undue reservation.

## Ethics statement

The studies involving human participants were reviewed and approved by Massey University. The patients/participants provided their written informed consent to participate in this study.

## Author contributions

XJ: conceptualization, data analysis, and manuscript writing. YP: supervision and manuscript revision. BH: manuscript revision and data collection. FH: manuscript revision. All authors contributed to the article and approved the submitted version.

## Footnotes

1
https://www.wjx.cn/

## Appendix

**APPENDIX TABLE 1:** The measurement items.

Constructs	No. of items	ID	Items	References
Confirmation	3	CO1	My experience with watching live streams was better than what I expected.	Bhattacherjee, 2001b
		CO2	I get the level of service I expect from stream-watching.	
		CO3	Overall, most of my expectations from watching live streams were confirmed.	
Perceived Value	3	PV1	Taking all the pros and cons into consideration, watching streams is beneficial to me.	Kim et al., 2007
		PV2	Comparing the benefits and sacrifices, watching streams is worthwhile for me.	
		PV3	Overall, watching streams gives me good value.	
Satisfaction	4	SA1	Watching streams makes me feel very satisfied.	Bhattacherjee, 2001b
		SA2	Watching streams gives me a sense of enjoyment.	
		SA3	Watching streams makes me feel very contented.	
		SA4	Watching streams makes me feel very delighted.	
Perceived Ease of Use	3	PEoU1	The use of live streaming to watch streams is clear and understandable.	Venkatesh and Davis, 1996
		PEoU2	I find it easy to watch streams.	
		PEoU3	I find it easy to do what I want to do in my use of live streaming to watch streams.	
Subjective Norms	3	SN1	People who are important to me want me to watch live streams.	Mathieson, 1991
		SN2	People who influence my behavior think I should watch live streams.	
		SN3	People whose opinions I value prefer me to watch live streams.	
Continuous Watching Intention	3	CWI1	I intend to continue watching streams rather than discontinue doing.	Bhattacherjee, 2001b
		CWI2	My intentions are to continue watching streams rather than to use any alternative means (watching TV).	
		CWI3	If I could, I would like to discontinue watching streams (reverse coded).	
